# Intrahepatic Type II Gall Bladder Perforation Secondary to Acute Acalculous Cholecystitis

**DOI:** 10.1155/2020/5435921

**Published:** 2020-03-25

**Authors:** Vimaleswaran Koculen, Umesh Jayarajah, Anil P. Ambawatte

**Affiliations:** ^1^Department of Surgery, Colombo South Teaching Hospital, Kalubowila, Colombo, Sri Lanka; ^2^Department of Surgery, National Hospital of Sri Lanka, Colombo, Sri Lanka

## Abstract

Gall bladder perforation is a rare but serious complication of acute cholecystitis. Gall bladder perforations commonly occur in patients with comorbidities and in association with gall stones. We report a rare occurrence of intrahepatic type II perforation of the gall bladder in a previously healthy elderly male with acalculous cholecystitis. Lack of early positive findings related to clinical assessment, laboratory workup, and imaging resulted in a challenging diagnosis. High degree of clinical suspicion and close monitoring in such patients is necessary to detect early deterioration and improve outcomes.

## 1. Introduction

Gall bladder perforation is a rare but serious complication of acute cholecystitis with an overall incidence between 3 and 15% [1]. Infrequently, clinical features are subtle during the onset with rapid clinical deterioration resulting in difficulties in the diagnosis and management of this condition [1, 2]. Gall bladder perforations commonly occur in patients with comorbidities and in association with gall stones [1, 2]. We report a rare occurrence of intrahepatic type II perforation of the gall bladder in a previously healthy elderly male with acalculous cholecystitis.

## 2. Case Presentation

A previously healthy 69-year-old man presented with a history of vague left sided abdominal pain with vomiting for one-day duration. His past medical and surgical history was unremarkable. He was afebrile and did not have any gastrointestinal or urinary symptoms on admission. On the second day of his illness, he experienced worsening of abdominal pain focused on the right upper quadrant with tenderness. His white blood cell count (6.16 × 10^9^/l) and C-reactive protein (less than 5 mg/dl) levels were normal. He had low platelets of 60 × 10^9^/l. His serum electrolytes and renal and liver functions were within normal limits. The abdominal ultrasound scan did not show any significant findings. He was managed symptomatically and was kept under observation.

However, on day 4, his clinical condition worsened with associated fever, jaundice, and reduced urine output. There was a tender hepatomegaly of 5 cm below the right costal margin. He also developed haemodynamic instability with a pulse rate of 126 per minute and systolic blood pressure of 90 mmHg. He developed acute respiratory distress syndrome and acute kidney injury with raised inflammatory markers, liver enzymes, and direct hyperbilirubinaemia. He was resuscitated, started on intravenous broad spectrum antibiotics, and was admitted to the intensive care unit for supportive management.

**Figure 1:**
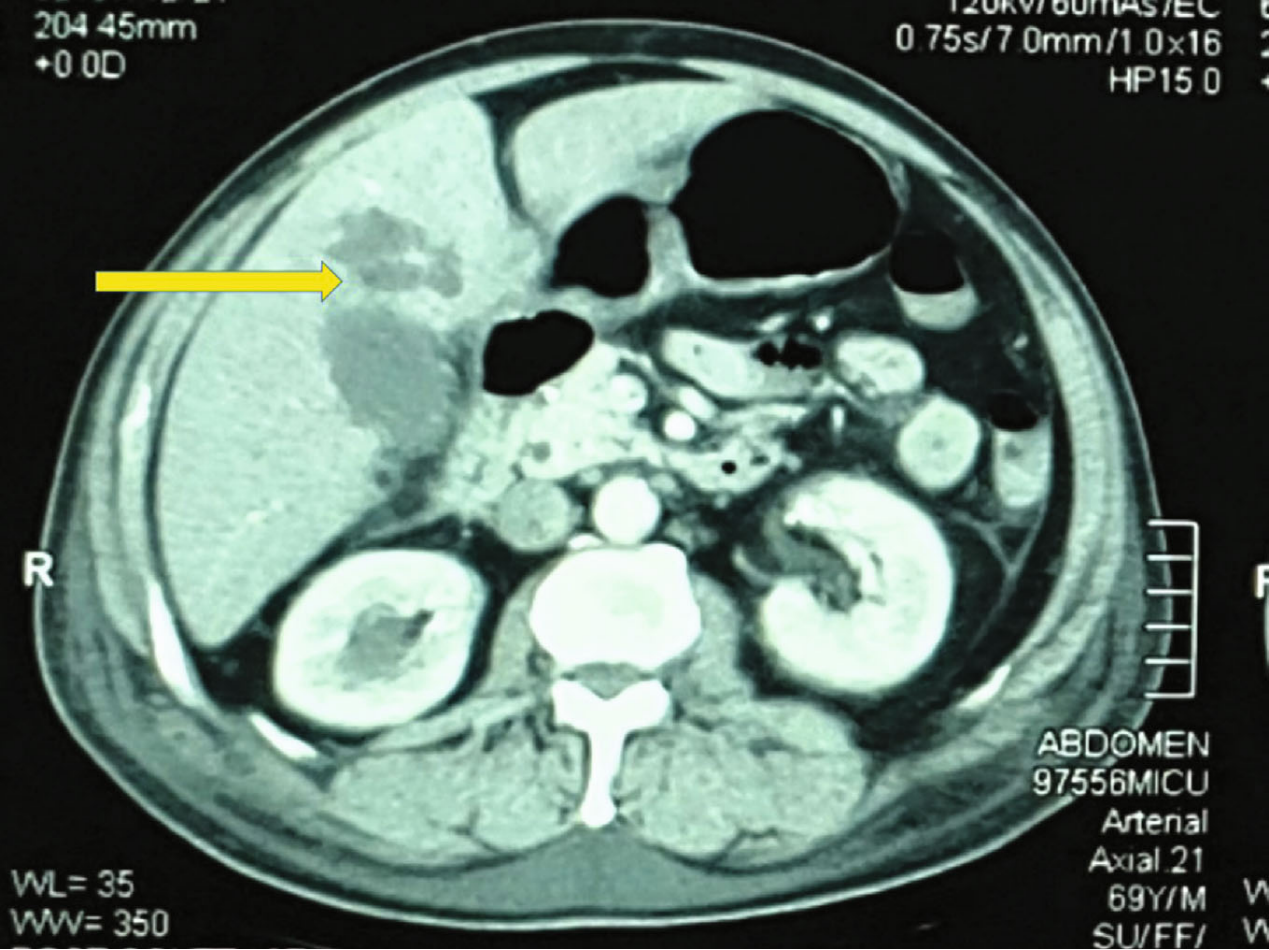
Contrast-enhanced computed tomography of the abdomen showing a 3 × 3 × 2 cm ill-defined, nonenhancing, hypodense area in segment V of the liver, compatible with a liver abscess.

**Figure 2:**
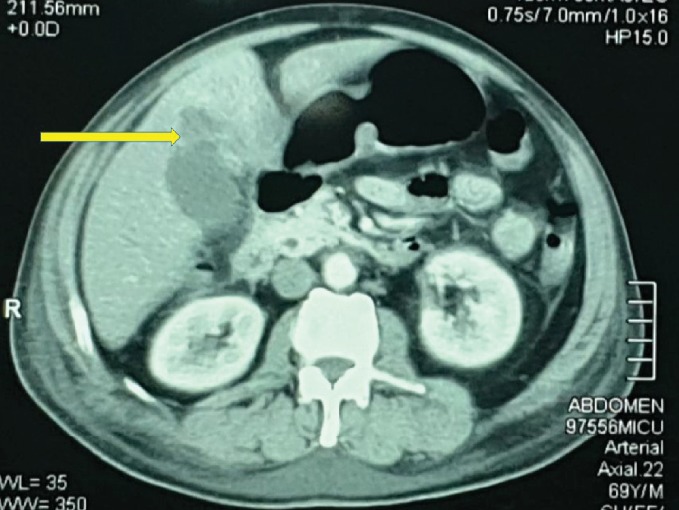
Contrast-enhanced computed tomography of the abdomen showing a 3 × 3 × 2 cm ill-defined, nonenhancing, hypodense area in segment V of the liver communicating with the fundus of the gall bladder.

The repeated abdominal ultrasonography revealed a small subhepatic fluid collection and hepatomegaly. There was a hypoechoic lesion in segment V. Contrast-enhanced computed tomography of the abdomen and chest revealed moderate hepatomegaly with a 3 × 3 × 2 cm ill-defined, nonenhancing, hypodense area in segment V, compatible with a liver abscess. The lesion was communicating with the fundus of the gall bladder. There was pericholecystic fluid suggestive of acute cholecystitis. There were no gall bladder calculi, and the biliary tree was normal. There was bilateral moderate pleural effusion with basal lung consolidation. Therefore, the findings were compatible with an intrahepatic abscess secondary to type II gall bladder perforation following acute acalculous cholecystitis (Figures 1 and 2).

He recovered with supportive management, and as the abscess was small and he was clinically improving, the abscess was managed conservatively. His blood culture and culture of the pleural fluid were negative. Intravenous antibiotics were continued for 4 weeks after consulting the microbiologist. The repeated ultrasonography showed considerable reduction in the size of the abscess. He was hospitalized for a total period of 5 weeks. Although he was offered an elective laparoscopic cholecystectomy, he later defaulted follow up.

## 3. Discussion

Gall bladder perforation is a rare complication of acute cholecystitis. The pathophysiology is explained by acute inflammation of the gall bladder with subsequent ischaemia and necrosis resulting in perforation of the gall bladder wall [1]. The fundus of the gall bladder is commonly perforated compared to other regions due to the relatively poor blood supply. This usually leads to spillage of the contents to the peritoneal cavity and biliary peritonitis which is known as acute type I perforation [3]. Rarely, the perforation may occur into the liver parenchyma leading to the formation of an intrahepatic abscess known as type II perforation. Long-standing cholecystoenteric fistula is known as type III perforation [1, 3].

Although uncomplicated acute cholecystitis occurs commonly in middle-aged females, gall bladder perforation is associated with a male preponderance, especially in the elderly [3]. Furthermore, it is associated with comorbidities such as type 2 diabetes, cirrhosis, malignancy, and immunodeficiency states. It is sometimes associated with initial mild, nonspecific symptoms followed by rapid deterioration due to sepsis, resulting in late diagnosis with serious consequences. Infrequently, the radiological investigations, laboratory tests, and clinical findings fail to establish the diagnosis. Furthermore, it is not uncommon to detect the gall bladder perforation only during surgery. Delay in diagnosis remains the most important factor leading to high morbidity and mortality [4].

Although the mortality rates were high in the past (approximately 40%), recent studies have shown reduced mortality rates of 12-16%, probably due to the advancement in the critical care [3]. Perforation may occur in a few days to several weeks after the onset of acute cholecystitis. Type I gall bladder perforations occur within a shorter duration as opposed to types II and III [2].

Abdominal ultrasonography remains the first line of investigation; however, the sensitivity is variable and it is operator dependent. Contrast-enhanced computed tomography of the abdomen is the gold standard in identifying a perforated gall bladder with sensitivities ranging from 81.3% to 88.2% [4]. Magnetic resonance cholangiopancreatography (MRCP) is similarly effective in the diagnosis of gall bladder perforation [4].

Early intervention and optimal critical care are necessary to improve outcome. Type I gall bladder perforation requires urgent open or laparoscopic cholecystectomy with abdominal lavage. Operative mortality rate in the acute setting ranges from 8 to 23% [5]. Late interventions are associated with increased morbidity, mortality, intensive care admissions, and long postoperative hospital stays. Furthermore, mortality rate rises to 90% in cases of late diagnosis and intervention [5]. Type II gall bladder perforations require cholecystectomy with drainage of abscess. Percutaneous transhepatic drainage of the gall bladder, and hepatic abscess remains an important minimally invasive approach followed by elective cholecystectomy. Type III gall bladder perforations often require cholecystectomy with excision of the cholecystoenteric fistula and suitable reconstruction [4].

There are several learning points in this reported case. This case describes a previously healthy elderly male presenting with nonspecific abdominal symptoms. The basic laboratory investigations and the ultrasonography were unremarkable with no gall bladder calculi. This leads to a delay in the diagnosis till he developed features of sepsis and organ dysfunction. High degree of clinical suspicion and close monitoring in such patients is necessary to detect early deterioration. Our patient recovered with supportive care and antibiotics. Developing an intrahepatic type II gall bladder perforation due to acute acalculous cholecystitis is a rare phenomenon.

## 4. Conclusion

We report a rare occurrence of intrahepatic type II perforation of the gall bladder in a previously healthy elderly male with acute acalculous cholecystitis. Lack of early positive findings related to clinical assessment, laboratory workup, and imaging resulted in a challenging and delayed diagnosis. High degree of clinical suspicion and close monitoring in such patients is necessary to detect early deterioration and improve outcomes.
